# Autonomous enhancement or emotional depletion? The dual-path mechanism of AI usage on employees’ work behavior

**DOI:** 10.3389/fpubh.2026.1695066

**Published:** 2026-03-18

**Authors:** Daokui Jiang, Yaru Liu, Xuan Gu

**Affiliations:** Business School, Shandong Normal University, Jinan, China

**Keywords:** AI usage, emotional exhaustion, innovative work behavior, sense of self-determination, work disengagement behavior

## Abstract

As a core driver of social progress, artificial intelligence (AI) has a far-reaching impact on employee behavior in the workplace. A dual-path model based on the SOR theory reveals the dual effects of AI usage. A four-stage longitudinal study analyzed data from 285 finance professionals, showing that AI usage can both boost innovative work behavior by enhancing sense of self-determination and predict work disengagement behavior via the emotional exhaustion elicited by AI-associated technostressors. Leaders’ AI symbolization plays a key moderating role, strengthening AI’s positive effect on sense of self-determination and lessening its negative impact on emotional exhaustion. This research offers theoretical and practical guidance for organizations to improve employee management strategies.

## Introduction

Propelled by the fintech wave, artificial intelligence (AI) is profoundly reshaping the operational models and service ecosystems of the financial industry. From robo-advisors to algorithmic risk management, AI technology is transforming the entire financial business chain with unprecedented reach and sophistication. While enhancing efficiency and optimizing costs, it is also reconfiguring employees’ work environments. On one hand, employees are freed from repetitive tasks, gaining greater autonomy to engage in innovation and decision-making (1). On the other hand, they are confronting psychological and behavioral challenges stemming from high-frequency human-machine interactions, algorithmic dependency, and skill obsolescence (2). Understanding how AI usage shapes employee behavior is therefore crucial for financial organizations seeking to maintain competitiveness during digital and intelligent transformation.

For the financial industry, which relies heavily on knowledge creation and client trust, employees’ innovative work behaviors and disengagement have become dichotomous indicators for measuring the effectiveness of organizational digital transformation. As artificial intelligence and automation technologies accelerate the replacement of routine, procedural tasks, the core competitiveness of financial institutions is shifting from standardized process execution to a reliance on employees’ capacity for proactive work that transcends their duties and creates high added value.

Existing research has explored factors influencing the aforementioned behaviors, primarily focusing on leadership styles, task attributes, and organizational environments (3–5). However, as artificial intelligence becomes deeply integrated into the financial sector, how it impacts employee attitudes and behaviors remains unclear, with a particular lack of systematic explanations for such AI-induced oppositional behaviors. Notably, the extant literature predominantly examines AI’s effects from a unidimensional perspective, either emphasizing its empowering potential or its disruptive consequences. This fragmented approach has yielded limited insight into how AI usage can simultaneously foster innovation and trigger disengagement—a duality that reflects its true “double-edged sword” nature. Moreover, there is scant research that systematically integrates both cognitive and emotional pathways to explain these divergent outcomes, nor has sufficient attention been paid to boundary conditions, such as leadership influence, that may shape these psychological processes. This theoretical gap hinders a holistic understanding of the complex interplay between AI and employee behavior in contemporary workplaces. Consequently, the primary objective of this research is to develop a dual-path theoretical model to systematically reveal the opposing mechanisms through which AI usage influences innovative work behavior and work disengagement behavior among financial industry employees.

This study offers significant contributions both theoretically and practically. Theoretically, by revealing the underlying pathways and boundary conditions through which AI usage influences employee behavior, it transcends simplistic dichotomous perspectives, thereby providing an integrated framework for understanding AI’s complex effects within organizations. Practically, the findings not only clarify AI’s micro-level operational mechanisms but also provide crucial insights for financial organizations. They equip these organizations to effectively guide technological empowerment and mitigate potential risks during digital and intelligent transformation, thereby advancing the goal of “technology for good” and fostering comprehensive employee development.

## Literature review and research hypotheses

### AI usage and employee innovative work behavior and work disengagement behavior

AI usage refers to the degree to which employees use artificial intelligence to achieve work goals (6). In this study, AI usage primarily refers to the behavior of financial industry employees using AI to perform work tasks, optimize business processes, and enhance decision-making abilities. We acknowledge that AI technologies vary in function and design, and different types of AI may influence employees through distinct psychological pathways. However, the present study examines AI usage as a generalized construct, emphasizing its overall intensity as a meaningful workplace stimulus. This approach allows us to explore the fundamental dual-path mechanisms that may underlie employee experiences with AI as an integrated technological suite, providing a foundational framework for future research to examine more nuanced, function-specific effects.

In the workplace, the use of AI is associated with significantly different behavioral outcomes. On one hand, it may promote innovative work behavior, which is defined as the voluntary generation, promotion, and implementation of new and beneficial ideas, processes, or products (7). It serves as a core driver for organizations to gain sustainable competitive advantage (8). On the other hand, AI usage may also trigger work disengagement behavior, characterized by psychological withdrawal and reduced investment in one’s work role (9). This form of disengagement can gradually undermine performance and wellbeing (10).

The Stimulus-Organism-Response (SOR) framework, provides a theoretical basis for understanding these opposing effects. It posits that external stimuli (S) influence internal cognitive and affective states of the organism (O), which in turn drive behavioral responses (R) (11). We propose that AI usage constitutes a significant workplace stimulus. However, employees’ interpretations of this stimulus can vary, leading to distinct psychological pathways and, consequently, opposite behaviors. The cognitive-empowerment path operates through cognitive appraisal. When AI usage enhances employees’ task control, provides efficacy-relevant feedback, or improves work coordination, it can fulfill basic psychological needs for autonomy and competence. Individuals with a stronger sense of autonomy, competence, and relatedness in relation to technology tend to hold a more positive attitude toward AI, thereby making them more likely to break away from routine norms and engage in innovative behaviors (12). Conversely, the affective-depletion path involves the induction of technostress by AI usage, exemplified by feelings of overload, invasion, or complexity (13). Managing this stress and its associated negative emotions, requires sustained emotional regulation. This process can deplete psychological resources and predict emotional exhaustion, a core dimension of burnout. To conserve their remaining resources, exhausted employees may cognitively and emotionally withdraw, manifesting as work disengagement.

Thus, AI usage can trigger both an empowering cognitive state and a depleting affective state, thereby explaining its dual association with innovative and disengagement behaviors. Most existing studies, however, have examined these effects in isolation. A critical gap remains in understanding the parallel mediating mechanisms that underlie this duality. Furthermore, social information processing theory suggests that the work environment provides cues which shape how employees interpret stimuli (14). As key social referents, leaders can influence these interpretations through their demonstrated attitudes and behaviors regarding AI, a concept known as leaders’ AI symbolization (15). This influence may potentially moderate the strength of the aforementioned psychological pathways. Building on these insights, this study develops an integrated dual-path model to systematically examine how AI usage influences employee behavior through sense of self-determination and emotional exhaustion, and how leaders’ AI symbolization may condition these relationships.

### The mediating role of sense of self-determination

Sense of self-determination is the intrinsic motivation employees feel when their needs for sense of self-determination, competence, and relatedness are met at work (16). When work conditions support these needs, individuals are more likely to internalize the value of their tasks, shifting from controlled, externally-driven motivation to more autonomous, self-endorsed forms of motivation. This internalization process enhances intrinsic motivation, which is a fundamental precursor to sustained, proactive, and innovative endeavors (17).

We propose that AI usage can be a significant work condition influencing the satisfaction of these basic needs, thereby fostering a sense of self-determination. The argument unfolds according to the three core needs of self-determination theory. Regarding the need for autonomy, AI can enhance perceived autonomy by taking over structured, rule-based components of tasks, such as data processing or preliminary analysis (18). This delegation allows employees greater discretion in how and when to engage with the core, judgment-intensive aspects of their work. By freeing employees from prescribed procedural steps, AI usage can facilitate a sense of volition and personal causation in work planning and execution. Concerning the need for competence, AI systems often provide immediate, data-driven feedback on task performance or outcomes (19). This feedback can clarify performance standards, illuminate areas for improvement, and offer a transparent gauge of progress. Successfully leveraging AI tools to achieve desired results can thus validate employees’ skills and efficacy, bolstering their belief in their sense of competence (20). As for the need for relatedness, it is critical to clarify that AI itself does not provide socio-emotional support. However, by automating routine coordination tasks and increasing process transparency, AI can reduce ambiguities in workflow handoffs. This clarity can facilitate smoother, more predictable interactions with colleagues, indirectly supporting a sense of connection and effective collaboration within the work unit (21). The satisfaction of relatedness thus stems from improved human-to-human coordination enabled by the technology, not from a relationship with the technology.

Importantly, the impact of AI on cognitive processes is nuanced. While it can offload routine mental labor and potentially free cognitive resources for more complex thinking, over-reliance may also pose risks, for instance, automation bias (22). In the context of this study, we focus on the motivational pathway. When AI usage is perceived as a tool that amplifies employees’ agency and effectiveness, meaning it supports autonomy and competence, it is likely to positively influence their sense of self-determination.

A heightened sense of self-determination, in turn, is a potent catalyst for innovative work behavior (23). To elaborate, the fulfillment of autonomy needs enables individuals to break free from organizational routines, proactively identify innovation opportunities, and take risks (24). An increase in competence strengthens individuals’ sense of efficacy in tackling innovative challenges, motivating them to direct their cognitive resources toward creative problem-solving (25). The establishment of relatedness needs fosters organizational trust, creating a psychologically safe environment that mitigates the psychological pressure associated with innovation failure (26). Empirical research consistently links self-determination to greater curiosity, exploration, and proactive problem-solving. These attributes are the essential components of innovation (27). Consequently, employees experiencing a strong sense of self-determination are more likely to direct their energy and cognitive resources toward generating, promoting, and implementing new and useful ideas at work.

Integrating these arguments within the SOR framework, AI usage serves as an environmental Stimulus (S). Employees’ cognitive appraisal of this stimulus, specifically the extent to which it is perceived to satisfy their basic psychological needs, shapes their internal Organism (O) state, namely their sense of self-determination. This empowered motivational state then drives the positive Response (R) of innovative work behavior. Based on the above analysis, this study proposes the following hypothesis:

H1: Sense of self-determination mediates the relationship between AI usage and employee innovative work behavior.

### The mediating role of emotional exhaustion

Emotional exhaustion is defined as a chronic state of psychological fatigue and depleted emotional resources resulting from excessive and prolonged job demands (28). It represents the core affective dimension of job burnout, characterized by feelings of being emotionally overextended, drained, and unable to recover. We contend that the introduction and intensive use of AI at work can be a significant source of such demands, primarily when viewed through the lens of technostress. Technostress refers to the stress individuals experience due to an inability to cope with demands associated with new information technologies (29). Prior research identifies several core dimensions of technostress highly relevant to AI contexts, including overload where employees are required to perform tasks faster and longer (30), intrusion where technology blurs work-life boundaries and demands constant connectivity (31), and complexity referring to the cognitive burden of learning and operating sophisticated, ever-evolving systems (32). AI usage can acutely trigger these dimensions, thereby depleting emotional resources.

Specifically, AI usage may predict emotional exhaustion through the following pathways rooted in technostress. The first is the pathway of threat and insecurity, often linked to overload and complexity. The perception that AI can automate complex tasks may evoke feelings of job insecurity or skill obsolescence (33). The constant pressure to “keep up” with AI’s capabilities and to prove one’s irreplaceable value creates a state of chronic anxiety and vigilance, which is emotionally draining. The second pathway involves the learning and adaptation burden, linked to complexity. AI systems often require continuous learning and adaptation. The need to acquire new technical skills, understand algorithmic outputs, and re-calibrate work processes imposes a significant cognitive load and demands sustained mental effort (34). This ongoing adaptation effort can be exhausting, especially when it feels obligatory and rapid. The third pathway is negative rumination and appraisal, a cognitive-affective process. Initial anxieties or negative experiences with AI can trigger persistent negative rumination. Employees may repetitively dwell on potential threats, personal inadequacy, or unfavorable future scenarios related to AI (35). This ruminative process amplifies and prolongs negative affective states, accelerating the depletion of emotional resources. The cumulative effect of these technostress-induced demands is the gradual erosion of an individual’s emotional resources, culminating in emotional exhaustion.

According to Conservation of Resources theory, individuals are motivated to acquire, retain, and protect their valued psychological and emotional resources (36). Emotional exhaustion represents a state in which these internal resources have become critically depleted. When facing such depletion, individuals act to prevent further resource loss, and thus work disengagement emerges as a key resource conservation strategy (37). Specifically, emotional exhaustion impairs self-regulatory capacity, making individuals more prone to adopting passive-avoidance coping strategies (38). Employees may attempt to alleviate emotional fatigue by reducing work-time investment, procrastinating on tasks, or mentally distancing themselves from the work context. These disengagement behaviors function as a functional adaptation: by withdrawing emotionally, cognitively, and behaviorally from the resource-draining source, individuals aim to preserve their remaining resources and prevent complete burnout.

Within AI-related work settings, the pressures of adapting to technological changes and managing human-machine collaboration conflicts impose additional emotional burdens on employees, thereby accelerating the depletion of their emotional resources (35). When stress induced by AI usage accumulates to a critical level and exacerbates emotional exhaustion, employees are more likely to resort to work disengagement as a self-protective mechanism to relieve pressure and halt the continuous drain on their resources.

Integrating this into the SOR framework, AI usage acts as a potent Stimulus (S) that can trigger technostress. This stress directly impacts the affective Organism (O) state, leading to emotional exhaustion. To cope with this depleted state and conserve resources, employees exhibit the Response (R) of work disengagement behavior. Based on the above analysis, this study proposes the following hypothesis:

H2: Emotional exhaustion mediates the relationship between AI usage and employee work-disengagement behavior.

### The moderating role of leaders’ AI symbolization

Leaders’ AI symbolization refers to leaders’ behavior of conveying their recognition and support for artificial intelligence technology through explicit signals such as words, actions, and objects (39). Examples include actively participating in AI discussions, utilizing AI tools, and sharing industry trends.

To understand how this symbolic behavior influences employees, we integrate Social Information Processing Theory into our core SOR framework. SIPT posits that individuals, when facing uncertain or ambiguous situations, rely on social cues from their environment, particularly from salient figures such as leaders, to interpret events, form attitudes, and guide their behavioral responses (40). We posit that leaders’ AI symbolization serves as a potent social cue that shapes the primary appraisal within the SOR sequence. Specifically, it influences how employees interpret the Stimulus (S) of “AI usage.” This appraisal, in turn, affects the resulting Organism (O) state. In essence, leaders’ AI symbolization moderates the first stage of the S-O link by coloring the meaning employees assign to the AI experience.

**Figure 1:**
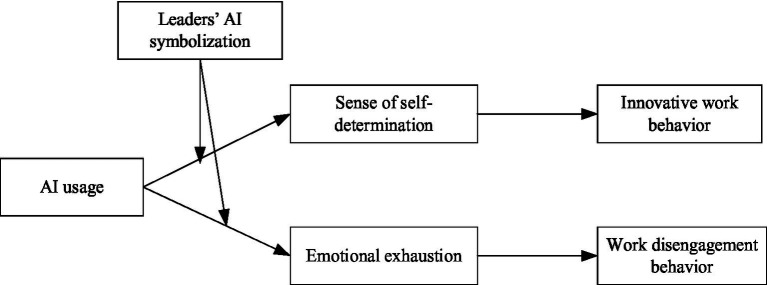
Theoretical model.

In a high-level leaders’ AI symbolization context, when leaders consistently and authentically symbolize support for AI, they send a signal that AI is a legitimate, valued, and empowering organizational tool (15). This cue facilitates a positive sense-making process. Employees are more likely to appraise AI usage as an opportunity for growth and enhancement. This appraisal amplifies the resource-gaining aspects of AI, thereby strengthening the positive relationship between AI usage and sense of self-determination. Concurrently, a strong leadership endorsement can buffer negative appraisals by framing challenges as temporary and supported, reducing perceptions of threat and isolation (41). Thus, it weakens the positive relationship between AI usage and emotional exhaustion (Figure 1).

Conversely, in a low-level leaders’ AI symbolization context, ambiguous, absent, or negative symbolic cues from leaders foster a sense of uncertainty and a lack of institutional support. Employees may interpret AI usage as an externally imposed demand, a surveillance mechanism, or a precursor to replacement (39). This negative sense-making process dampens the perception of AI as an empowering resource, weakening its potential to foster a sense of self-determination. Moreover, without the reassuring frame provided by leadership support, the inherent demands and threats of AI are more likely to be appraised as overwhelming and unsupported, thereby strengthening the link between AI usage and emotional exhaustion. Based on this, the study proposes:

H3a: Leaders’ AI symbolization moderates the relationship between AI usage and sense of self-determination. The higher the degree of leaders’ AI symbolization, the stronger the positive impact of AI usage on sense of self-determination.

H3b: Leaders’ AI symbolization moderates the relationship between AI usage and emotional exhaustion. The higher the degree of leaders’ AI symbolization, the weaker the positive impact of AI usage on emotional exhaustion.

Based on the above, this study further proposes that the mediating effects of sense of self-determination and emotional exhaustion between AI usage and employee behavior are influenced by leaders’ AI symbolization. Specifically, when leaders’ AI symbolization is high, leaders’ positive attitudes and supportive behaviors toward AI can enhance employees’ sense of self-determination during AI usage. Employees in this state are more likely to focus their energy and attention on innovative work, thereby actively exploring ways to use AI for innovation. On the other hand, when leaders’ AI symbolization is low, employees are more likely to feel stressed due to lack of support and guidance while using AI, which can trigger emotional exhaustion. In such negative emotional states, employees are more inclined to avoid work, leading to an increase in work disengagement behaviors. Based on the above analysis, this study proposes the following hypotheses:

H4a: Leaders’ AI symbolization positively moderates the mediating role of sense of self-determination between AI usage and employee innovative work behavior. That is, the higher the level of leaders’ AI symbolization, the stronger the mediating effect of sense of self-determination in the relationship between AI usage and employee innovative work behavior.

H4b: Leaders’ AI symbolization negatively moderates the mediating role of emotional exhaustion between AI usage and employee work disengagement behavior. That is, the higher the level of leaders’ AI symbolization, the weaker the mediating effect of emotional exhaustion in the relationship between AI usage and employee work disengagement behavior.

## Research design

### Research procedure and sample characteristics

This study uses a longitudinal design to gather data from China’s financial sector. Participants who met the criteria were recruited and filtered via Credamo, an online survey platform, and were sent questionnaires. The survey was carried out in two steps: a pilot survey and the main survey. In the pilot phase, a small-scale test was done on Credamo to make sure the measurement items were clear and complete. The questionnaire began with an explanation of the survey’s purpose, process, and requirements, along with a promise that the data would only be used for academic purposes and that personal information would be kept private. Then, participants read a background text that described the concepts and characteristics of artificial intelligence and answered a screening question: “Do you use AI in your daily work?” Those who answered “yes” continued with the survey, while those who answered “no” were automatically stopped. One hundred and fifty completed questionnaires were collected in the pilot survey. One hundred and forty-six passed the screening question. After removing 20 invalid ones due to unreasonable answering time, logical inconsistencies, or patterned responses, 126 valid questionnaires remained (an effective recovery rate of 84%). Based on this sample, the scale’s reliability and validity were analyzed, and some items were adjusted to ensure the measurement tool’s reliability and validity.

The formal survey used a four-phase design, adapted from Burhan (42) with a three-week interval between each measurement. The three-week spacing is appropriate for capturing the psychological processes examined in this study because changes in employees’ autonomy perceptions and emotional states generally develop over a period of several weeks of ongoing work experience. A shorter interval may not allow these processes to accumulate sufficiently to be observed, whereas overly long intervals would greatly increase sample attrition in a multi-wave survey. Studies in organizational behavior and information systems frequently employ intervals between two and four weeks when investigating similar short-term psychological mechanisms. Using a three-week interval therefore offers a balanced choice that aligns with established empirical practice and provides an appropriate temporal window for detecting meaningful changes in the variables of interest.

To ensure temporal clarity in the causal ordering of variables, all constructs in the proposed model follow a T_*n* − 1_ → T*_n_* → T_*n* + 1_ alignment structure. AI usage was measured at T_1_ and treated as the exogenous stimulus. The two psychological mechanisms—sense of self-determination and emotional exhaustion—were measured at T_2_ and T_3_ respectively, representing temporally lagged organism states that develop after initial exposure to AI usage. Behavioral outcomes, including innovative work behavior and work disengagement, were measured at T_4_ to capture employees’ subsequent responses. This arrangement ensures that predictors temporally precede mediators, and mediators temporally precede outcomes, thereby reducing simultaneity concerns and enhancing the interpretability of the temporal causal chain. In addition, the spacing between adjacent waves was held constant, allowing the T_*n* − 1_ → T*_n_* → T_*n* + 1_ sequence to reflect comparable time intervals and supporting a consistent developmental logic across the multi-wave design.

To ensure quality, attention-check questions (e.g., “Strongly disagree for this item”) were added. Responses not matching the specified option were deemed invalid. In the T_1_ phase, 492 people took part. One was stopped after answering “No” to the AI-usage question. The remaining 491 provided demographic details and measured key variables. 383 valid responses were received (77.85% valid response rate). At T_2_, 383 T1 respondents were re-invited to measure the same variables again. 347 valid responses were collected (90.60% valid response rate). In the T_3_ phase, 347 T2 respondents were invited back. 315 valid responses were recovered (90.78% valid response rate). At T_4_, 315 T3 respondents were asked to participate again. 288 valid responses were obtained (90.48% valid response rate). Finally, after excluding questionnaires with obvious response patterns and those that could not be matched across all phases, 285 valid questionnaires were left.

In the final sample, 73.7% were female. In terms of age distribution, the majority of employees (47.7%) fell within the 26–35 age bracket, while only a small proportion (4.9%) were aged 46 or above. Regarding educational background, most participants held a bachelor’s degree (66%), with 29.1% having obtained a master’s degree or higher, and just 4.9% having an associate degree or lower. As for work experience, 62.8% of the employees had been in the workforce for five years or more, and 9.1% had less than one year of work experience.

### Variables measurement

The study utilized well-established scales, employing a 7-point Likert-type scale (1 = “Strongly disagree” to 7 = “Strongly agree”).

*AI usage* was measured using a scale developed by Tang et al. (2022) (6), consisting of 3 items (e.g., “I use AI for most of my work”). Its Cronbach’s *α* values over four measurements were 0.805, 0.753, 0.784, and 0.776.

*Sense of self-determination* was measured using a scale by Terblanche (2024) (43), with 3 items (e.g., “After using AI, I feel competent and efficient”). The Cronbach’s *α* values were 0.753, 0.792, 0.808, and 0.816.

*Emotional exhaustion* was measured with a scale from Watkins et al. (2015) (44), including 3 items (e.g., “Work exhausts me”). Its Cronbach’s *α* values were 0.881, 0.897, 0.883, and 0.903.

*Innovative work behavior* was assessed using a scale developed by Scott and Bruce (1994) (45), with 4 items (e.g., “I develop proper plans to implement new ideas”). The Cronbach’s *α* values were 0.747, 0.775, 0.789, and 0.802.

*Work disengagement behavior* was measured via a scale from Demerouti et al. (2003) (46), with 4 items (e.g., “I talk negatively about my work more and more”). The Cronbach’s *α* values were 0.929, 0.939, 0.937, and 0.937.

*Leaders’ AI symbolization* was assessed via a scale from He et al. (2023) (39), with 4 items (e.g., “My leader shows interest in AI”). The Cronbach’s *α* coefficient for this variable was 0.857.

### Data analysis methods

To test the hypotheses of this study, we employed a hierarchical linear model to analyze longitudinal data collected across four waves. This approach effectively handles the nested structure of within-person variables (AI usage, sense of self-determination, etc.) and between-person variables (leaders’ AI symbolization), while respecting the temporal ordering of the variables. All analyses were conducted using Mplus 8.3 with Bayesian estimation methods to obtain robust model fit and address missing data.

## Data analysis and hypothesis testing

### Descriptive statistics and confirmatory factor analysis

The variables’ mean, standard deviation, and correlation coefficient are in Table 1. A multilevel confirmatory factor analysis was conducted on all data comprising five variables: AI usage, sense of self-determination, emotional exhaustion, innovative work behavior, work disengagement behavior and leaders’ AI symbolization. The results showed that the fit indices of the six-factor model were optimal (*χ*^2^ = 457.239; df = 283; CFI = 0.967; TLI = 0.959; RMSEA = 0.023; SRMR_within_ = 0.032; SRMR_between_ = 0.050). This indicates that the factor structure aligns with expectations, the variables demonstrate high discriminant validity, and the model meets the requirements of the study. Although this study adopted a longitudinal design to mitigate common method bias (CMB), Harman’s single-factor test (47) was still used to check for systematic interference. The first principal component’s variance accounted for at each time point (T_1_: 33.168%; T_2_: 35.552%; T_3_: 33.828%; T_4_: 36.058%) was below the 40% threshold, indicating no severe CMB issue (Table 2).

**Table 1:** Means, standard deviations, and correlations.

Variables	M	SD	1	2	3	4	5	6	7	8	9	10	11	12
1. T_1_ AIU	5.083	1.045	1											
2. T_2_ AIU	5.305	0.952	0.688**	1										
3. T_3_ AIU	5.360	0.991	0.748**	0.836**	1									
4. T_4_ AIU	5.359	0.960	0.741**	0.773**	0.842**	1								
5. T_1_ SD	5.439	0.759	0.633**	0.485**	0.518**	0.501**	1							
6. T_2_ SD	5.568	0.835	0.555**	0.640**	0.597**	0.607**	0.662**	1						
7. T_3_ SD	5.557	0.831	0.545**	0.583**	0.612**	0.583**	0.594**	0.638**	1					
8. T_4_ SD	5.606	0.831	0.559**	0.580**	0.592**	0.638**	0.620**	0.734**	0.788**	1				
9. T_1_ EE	5.030	1.348	0.281**	0.278**	0.310**	0.246**	0.328**	0.350**	0.259**	0.307**	1			
10. T_2_ EE	5.030	1.395	0.303**	0.313**	0.331**	0.269**	0.343**	0.380**	0.294**	0.354**	0.816**	1		
11. T_3_ EE	5.068	1.351	0.303**	0.321**	0.339**	0.264**	0.336**	0.372**	0.296**	0.349**	0.824**	0.855**	1	
12. T_4_ EE	5.025	1.404	0.313**	0.312**	0.318**	0.269**	0.391**	0.415**	0.328**	0.418**	0.800**	0.824**	0.852**	1
13. T_1_ IWB	5.600	0.718	0.547**	0.527**	0.540**	0.539**	0.551**	0.572**	0.422**	0.512**	0.354**	0.353**	0.371**	0.348**
14. T_2_ IWB	5.618	0.763	0.460**	0.493**	0.483**	0.465**	0.482**	0.510**	0.369**	0.470**	0.476**	0.451**	0.455**	0.452**
15. T_3_ IWB	5.581	0.805	0.448**	0.515**	0.490**	0.472**	0.472**	0.508**	0.437**	0.540**	0.413**	0.388**	0.420**	0.433**
16. T_4_ IWB	5.615	0.776	0.496**	0.486**	0.486**	0.480**	0.523**	0.493**	0.424**	0.538**	0.374**	0.367**	0.398**	0.433**
17. T_1_ WDB	4.148	1.693	0.097	0.140*	0.113	0.096	0.200**	0.174**	0.129*	0.181**	0.274**	0.220**	0.189**	0.217**
18. T_2_ WDB	4.159	1.752	0.109	0.157**	0.118*	0.113	0.184**	0.158**	0.140*	0.196**	0.251**	0.282**	0.204**	0.207**
19. T_3_ WDB	4.117	1.772	0.103	0.187**	0.143*	0.144*	0.143*	0.159**	0.153**	0.190**	0.246**	0.231**	0.239**	0.207**
20. T_4_WDB	4.160	1.746	0.122*	0.195**	0.166**	0.161**	0.169**	0.186**	0.137*	0.204**	0.227**	0.223**	0.176**	0.215**
21. LAS	5.407	1.010	0.525**	0.528**	0.552**	0.599**	0.470**	0.603**	0.611**	0.710**	0.318**	0.344**	0.325**	0.362**
22. Gender	1.74	0.441	0.012	0.030	−0.013	−0.023	−0.029	−0.019	0.001	0.023	0.051	0.114	0.064	0.088
23. Age	2.20	0.795	0.001	0.018	0.007	−0.013	0.080	0.127*	0.092	0.111	0.278**	0.228**	0.234**	0.299**
24. Education	3.24	0.554	0.021	−0.014	−0.005	0.024	0.111	0.055	−0.030	0.013	0.096	0.102	0.077	0.089
25. Tenure	3.62	1.304	0.021	0.022	0.015	−0.002	0.152*	0.161**	0.126*	0.156**	0.321**	0.279**	0.254**	0.324**

Variables	13	14	15	16	17	18	19	20	21	225	23	24	25
13. T_1_ IWB	1												
14. T_2_ IWB	0.682**	1											
15. T_3_ IWB	0.727**	0.719**	1										
16. T_4_ IWB	0.702**	0.750**	0.799**	1									
17. T_1_ WDB	0.146*	0.144*	0.180**	0.149*	1								
18. T_2_ WDB	0.120*	0.135*	0.168**	0.139*	0.891**	1							
19. T_3_ WDB	0.125*	0.167**	0.160**	0.161**	0.897**	0.892**	1						
20. T_4_ WDB	0.133*	0.169**	0.163**	0.124*	0.875**	0.898**	0.901**	1					
21. LAS	0.465**	0.463**	0.463**	0.449**	0.204**	0.213**	0.208**	0.216**	1				
22. Gender	−0.150*	−0.007	−0.086	−0.066	0.022	0.088	0.030	0.076	0.052	1			
23. Age	0.151*	0.089	0.145*	0.062	0.122*	0.103	0.066	0.083	0.113	−0.050	1		
24. Education	0.124*	0.091	0.050	0.109	0.060	0.028	0.027	−0.008	0.016	−0.034	−0.035	1	
25. Tenure	0.206**	0.168**	0.189**	0.132*	0.122*	0.099	0.067	0.087	0.103	−0.033	0.756**	−0.066	1

*N* = 285. *p < 0.05. **p < 0.01. ***p < 0.001. AIU = AI usage, LAS = leaders’ AI symbolization, SD = sense of self-determination, EE = emotional exhaustion, IWB = innovative work behavior, WDB = work disengagement behavior. T_1_ = Time1, T_2_ = Time 2, T_3_ = Time 3, T_4_ = Time 4.

**Table 2:** The confirmatory factor analysis.

Model	*χ* ^2^	df	*χ*^2^/df	CFI	TLI	RMSEA	SRMR_with_	SRMR_between_
6 factors (AIU; SD; EE; IWB; WDB; LAS)	457.239	283	1.616	0.967	0.959	0.023	0.032	0.050
5 factors (AIU + SD; EE; IWB; WDB; LAS)	622.288	292	2.131	0.937	0.925	0.031	0.036	0.050
4 factors (AIU + SD; EE + WDB; IWB; LAS)	1,513.525	299	5.068	0.767	0.731	0.060	0.045	0.174
3 factors (AIU + SD + IWB, EE + WDB; LAS)	1,782.117	304	5.862	0.717	0.678	0.065	0.056	0.177
2 factors (AIU + EE + WDB; SD + IWB + LAS)	3,289.241	314	10.475	0.430	0.372	0.091	0.079	0.210
1 factor (AIU + SD + EE + IWB + WDB + LAS)	4,260.821	329	12.951	0.247	0.208	0.102	0.106	0.303

*N* = 285. AIU = AI usage, SD = sense of self-determination, EE = emotional exhaustion, IWB = innovative work behavior, WDB = work disengagement behavior.

### Longitudinal measurement invariance testing

As shown in Table 3, the test results for the difference in fitting indices between adjacent pattern models are less than the critical value (ΔCFI ≤ 0.01) (48). Thus, measurement invariance was confirmed, indicating reliable repeated measurements over four times for AI usage, sense of self-determination, emotional exhaustion, innovative work behavior, and work disengagement behavior, scales.

**Table 3:** Measurement equivalence test.

Model	*χ* ^2^	*df*	TLI	CFI	RMSEA	SRMR	△*χ*^2^	△*df*	△RMSEA	△CFI
Configural invariance	237.453	134	0.960	0.972	0.052	0.054	–	–	–	–
Weak invariance	323.087	146	0.961	0.970	0.065	0.064	85.634	12	0.013	−0.002
Strong invariance	386.525	161	0.954	0.961	0.070	0.068	63.4438	15	0.005	−0.009

### Hypothesis testing

The hypothesis testing employed hierarchical linear regression models constructed in Mplus 8.3. Initial null model tests checked if the within-individual variability of the dependent variables showed significant between-group differences. The results indicated significant between-group variance for innovative work behavior and work disengagement behavior, with ICC1 values of 0.729 and 0.894, respectively, both above the 0.059 threshold, thus supporting subsequent tests. Table 4 displays the analysis results of the hierarchical linear regression models.

**Table 4:** Results of analyses of hierarchical linear regression models.

Variables	IWB(T_*n* + 1_)		WDB(T_*n* + 1_)		SD(T*_n_*)		EE(T*_n_*)	
	Model 1	Model 2	Model 3	Model 4	Model 5	Model 6	Model 7	Model 8
Intercept	4.285***	3.401***	3.439***	2.627***	3.280***	4.333***	2.575***	2.457***
Time	0.000	0.009	−0.028	−0.032	−0.099*	0.019	−0.009	−0.030
Independent variable								
AIU(T_*n*-1_)	0.157***	0.128***	0.133**	0.112*	0.400***	0.371***	0.211***	0.148*
Mediator								
SD(T*_n_*)		0.197***						
EE(T*_n_*)				0.256***				
Moderator								
LAS						0.321***		0.548**
Interaction								
AIU(T_*n*-1_) × LAS						0.036*		−0.070*
Control variables								
Gender	−0.131	−0.130	−0.014	−0.067	−0.027	−0.008	0.196	0.318*
Age	−0.045	−0.044	−0.029	−0.037	0.005	−0.047	0.046	0.112
Education	0.124*	0.117	0.073	0.042	0.028	0.017	0.117	0.236*
Tenure	0.117**	0.099*	0.137	0.090	0.083*	0.064*	0.179**	0.219**
Variance components								
Within-group variation	0.145***	0.149***	0.295***	0.316***	0.318***	0.217***	0.276***	0.368***
Between-group variation	0.378***	0.321***	1.773***	1.492***	0.154***	0.148***	0.985***	0.960***

*N* = 285. *p < 0.05. **p < 0.01. ***p < 0.001. AIU = AI usage, LAS = leaders’ AI symbolization, SD = sense of self-determination, EE = emotional exhaustion, IWB = innovative work behavior, WDB = work disengagement behavior.

#### Mediating effect test

Table 4 presents the results of the hierarchical linear regression analysis. Regarding the positive path, Model 1 showed that AI usage (T_*n*-1_) positively predicted innovative work behavior (T_*n* + 1_) (*β* = 0.157, *p* < 0.001). After introducing the mediator in Model 2, AI usage (T_*n*-1_) remained a significant predictor of innovative work behavior (T_*n* + 1_) (*β* = 0.128, *p* < 0.001), and sense of self-determination (T*_n_*) was a positive predictor of the outcome (*β* = 0.197, *p* < 0.001). Furthermore, Model 5 demonstrated that AI usage (T_*n*-1_) significantly predicted sense of self-determination (T*_n_*) (*β* = 0.400, *p* < 0.001). These results provide initial support for H1, suggesting a mediating role of sense of self-determination. Concerning the negative path, Model 3 indicated that AI usage (T_*n*-1_) positively predicted work disengagement behavior (T_*n* + 1_) (*β* = 0.133, *p* < 0.01). When emotional exhaustion was added in Model 4, AI usage (T_*n*-1_) remained a significant predictor of work disengagement behavior (T_*n* + 1_) (*β* = 0.112, *p* < 0.05), and emotional exhaustion (T*_n_*) was a strong positive predictor (*β* = 0.256, *p* < 0.001). Model 7 confirmed that AI usage (T_*n*-1_) also significantly predicted emotional exhaustion (T*_n_*) (*β* = 0.211, *p* < 0.001). Together, these findings support H2, indicating emotional exhaustion as a mediator.

To further test the mediating effect hypotheses, the Markov chain Monte Carlo (MCMC) method in Bayesian analysis was applied. As shown in Table 5, the indirect effect of AI usage (T_*n*-1_) on innovative work behavior (T_*n* + 1_) through sense of self-determination (T*_n_*) was significant (*β* = 0.092, SE = 0.018, 95% CI [0.056, 0.127]), as was the indirect effect on work disengagement behavior (T_*n* + 1_) through emotional exhaustion (T*_n_*) (*β* = 0.124, SE = 0.028, 95% CI [0.072, 0.182]). Thus, H1 and H2 are further supported.

**Table 5:** Results of mediating effect analysis.

Influence path	Coefficients	Standard error	95% confidence interval	
			Lower limit	Upper limit
AIU(T_*n*-1_) → SD(T*_n_*) → IWB(T_*n* + 1_)	0.092***	0.018	0.056	0.127
AIU(T_*n*-1_) → EE(T*_n_*) → WDB(T_*n* + 1_)	0.124***	0.028	0.072	0.182

*N* = 285. *p < 0.05. **p < 0.01. ***p < 0.001. AIU = AI usage, SD = sense of self-determination, EE = emotional exhaustion, IWB = innovative work behavior, WDB = work disengagement behavior.

#### Moderating effect test

**Figure 2:**
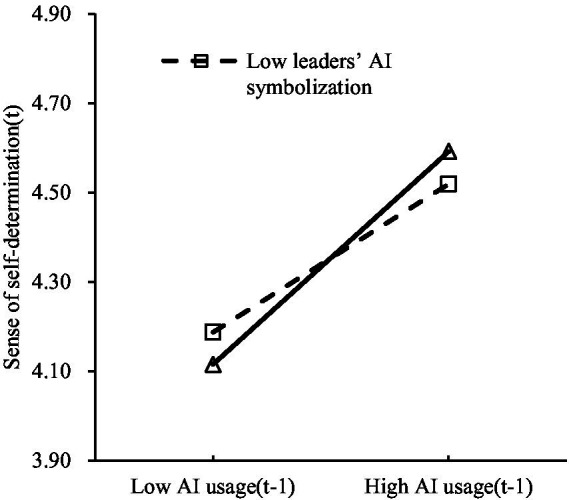
Leaders’ AI symbolization moderates the relationship between AI usage (T_*n*-1_) and sense of self-determination (T*_n_*).

**Figure 3:**
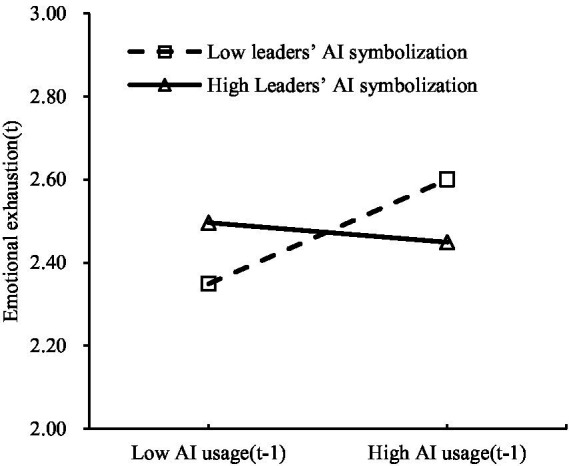
Leaders’ AI symbolization moderates the relationship between AI usage (T_*n*-1_) and emotional exhaustion (T*_n_*).

Table 4 model 6 shows that the interaction term of AI usage (T_*n*-1_) and leaders’ AI symbolization significantly affects sense of self-determination (T*_n_*) (*β* = 0.036, *p* < 0.05), indicating a significant moderation effect of leaders’ AI symbolization on the relationship between AI usage (T_*n*-1_) and sense of self-determination (T*_n_*). Table 4 model 8 reveals that the interaction term of AI usage (T_*n*-1_) and leaders’ AI symbolization significantly impacts emotional exhaustion (T*_n_*) (*β* = −0.070, *p* < 0.05), showing a significant moderation effect of leaders’ AI symbolization on the link between AI usage (T_*n*-1_) and emotional exhaustion (T*_n_*). To better illustrate these moderation effects, moderation plots were created (see Figures 2, 3).

As shown in Figure 2, the higher the level of leaders’ AI symbolization, the stronger the positive impact of AI usage (T_*n*-1_) on sense of self-determination (T*_n_*). Conversely, this positive effect weakens when leaders’ AI symbolization is low. Thus, H3a is verified. Figure 3 indicates that when leaders’ AI symbolization is low, AI usage (T_*n*-1_) significantly increases emotional exhaustion (T*_n_*). The opposite is also true, which confirms H3b.

#### Moderated mediation analysis

This study further tested the moderated mediation model using the Markov chain Monte Carlo (MCMC) method. The results are shown in Table 6. For the sense of self-determination path, when leaders’ AI symbolization was low, the indirect effect of AI usage (T_*n*-1_) on employee innovative work behavior (T_*n* + 1_) via sense of self-determination (T*_n_*) was 0.039 (95% CI [0.020, 0.063], which does not include 0). When leaders’ AI symbolization was high, this indirect effect increased to 0.080 (95% CI [0.050, 0.113], which also does not include 0). The difference between the two groups was 0.040 (95% CI [0.025, 0.057], which does not include 0), indicating a significant difference. Therefore, H4a is supported.

**Table 6:** Results of the moderated mediation effect test.

Mediator variable	LAS	Coefficients	Standard error	95% confidence interval
SD(T*_n_*)	Low level (−1 SD)	0.039***	0.011	[0.020, 0.063]
	High level (+1 SD)	0.080***	0.016	[0.050, 0.113]
	High–Low difference	0.040***	0.008	[0.025, 0.057]
EE(T*_n_*)	Low level (−1 SD)	0.080***	0.022	[0.040, 0.129]
	High level (+1 SD)	0.036***	0.018	[0.007, 0.076]
	High–Low difference	−0.043**	0.014	[−0.073, −0.020]

*N* = 285. *p < 0.05. **p < 0.01. ***p < 0.001. LAS = leaders’ AI symbolization, SD = sense of self-determination, EE = emotional exhaustion.

For the emotional exhaustion path, when leaders’ AI symbolization was low, the indirect effect of AI usage (T_*n*-1_) on work disengagement behavior (T_*n* + 1_) via emotional exhaustion (T*_n_*) was 0.080 (95% CI [0.040, 0.129], which does not include 0). When leaders’ AI symbolization was high, this indirect effect was 0.036 (95% CI [0.007, 0.076], which also does not include 0). The difference between the two groups was −0.043 (95% CI [−0.073, −0.020], which does not include 0), indicating a significant difference. Thus, H4b is supported.

## Discussion

Building on the SOR framework, this study developed a dual-path model to explain how AI usage influences employee behavior. Results show that AI usage promotes innovative work behavior by enhancing employees’ sense of self-determination, while it also facilitates work disengagement through increased emotional exhaustion. Leaders’ AI symbolization moderates these pathways, modestly strengthening the mediating role of self-determination and weakly attenuating the mediating role of emotional exhaustion. Although statistically significant, these moderating effects are not dominant, indicating that leaders’ AI symbolization represents one meaningful yet partial boundary condition in managing AI’s dual effects.

A critical boundary condition must be underscored. The AI functions examined here are distinct in nature from the complex socio-emotional and clinical support required for employee wellbeing. The latter must remain the responsibility of qualified human professionals such as organizational psychologists, counselors, and skilled managers. This research does not advocate replacing human relational support with technology; rather, it focuses on how task-supportive AI can augment human work, with the goal of informing practices that preserve and enhance the essential human aspects of organizational life.

### Theoretical implications

Firstly, this study delves into AI usage’s impact on employees’ psychology and behavior, enriching micro-level AI research and it provides a new contextualized perspective for understanding organizational behavior research in the financial industry during its digital and intelligent transformation. Prior studies on employee innovative work behavior and work disengagement behavior mainly center on conventional work settings, with limited attention to the situational changes brought by intelligent technology. By constructing a model of AI usage’s influence on employee work behavior, this study uncovers the internal mechanisms and expands the research context to the field of AI application, addressing the theoretical one-sidedness in existing research.

Secondly, within the SOR theory framework, this study explores the dual paths of AI’s impact on employees through cognitive evaluation and emotional experience. Earlier research often analyses AI’s impact on employee behavior from a single perspective. Yet, AI, with its enabling and intrusive features, has contradictory effects. By establishing a parallel mediating model of sense of self-determination and emotional exhaustion, this study reveals employees’ varying behavioral responses to AI usage due to cognitive motivation and emotional differences. It contributes to a deeper understanding of how AI usage influences employee work behaviors within the high-pressure, high-compliance context of China’s financial industry, thereby establishing a foundation for subsequent contextualized comparative research.

Thirdly, this study examines the moderating role of leadership in AI symbolization and enriches the boundary conditions of AI usage’s influence on employee behavior. Rooted in social information processing theory, individuals interpret environmental cues to form situational cognition and emotional responses, driving behavioral decisions. This study innovatively applies the theory to AI-usage scenarios, viewing leaders’ AI symbolization as a key social information source that reshapes employees’ cognitive evaluation and emotional experience of AI. This finding confirms the theory’s explanatory power in tech-change contexts, broadens leadership theory from a tech-application perspective, and offers a new theoretical lens for understanding leadership mechanisms in human-machine collaboration settings.

### Practical implications

Firstly, at the organizational level: establish an intelligent and human-centric management system to systematically manage the dual impact of AI. Financial institutions should first abandon the simplistic mindset of technological determinism and fully recognize that AI usage simultaneously generates both enabling and draining effects on employees’ psychology and behavior. To this end, enterprises need to commit to building an organic human-machine collaborative system: while leveraging AI to enhance efficiency and liberate employees from repetitive tasks, they must simultaneously establish regular skills training programs and career development support systems to alleviate employees’ anxiety about competence and insecurity. Furthermore, organizations should incorporate employee psychological wellbeing as a dimension in evaluating technological effectiveness. By establishing mental health monitoring and support mechanisms, they can proactively intervene in emotional exhaustion triggered by technostress, fundamentally balancing technological efficiency with organizational humanistic care.

Secondly, at the leadership level: leverage the “symbolization” role to become both an enabler and a buffer for employees during their technological adaptation process. In the intelligent transformation, the role of leaders is crucial. They should not merely be implementers of technology, but rather demonstrators and sense-makers of human-machine collaboration. Leaders need to clearly communicate the value and methods of technological empowerment through their own active use of AI, while proactively defining the boundaries of human-AI responsibilities to reduce employees’ role ambiguity. More importantly, leaders should establish open and inclusive communication channels to timely identify psychological pressures and emotional needs that emerge during employees’ adaptation process, providing prompt emotional support. Through such “symbolizing” leadership, the challenges brought by AI can be effectively transformed into shared opportunities for team innovation, guiding technological transformation toward sustainable development.

Thirdly, at the employee level: shift from passive adaptation to active mastery, achieving symbiotic development with AI. Employees themselves should maintain clear awareness of the dual nature of AI and proactively engage in self-adjustment to harness the technology. Specifically, employees should transform passivity into initiative by actively participating in learning AI tools, with the goal of mastering advanced work models involving AI-assisted decision-making rather than remaining at the level of passively executing commands. In their work, they should consciously plan the rhythm of human-machine collaboration—for instance, designating specific time blocks for AI to handle procedural tasks—to avoid falling into a state of continuous technical stress and to preserve focused cognitive space for creative work. Ultimately, by actively managing their relationship with technology, employees need to continuously enhance their sense of self-determination, effectively maintain the psychological resources necessary for innovation, and discover a sustainable growth trajectory within human-AI collaboration.

### Limitations

First, although the multi-wave design helps reduce concerns about common method bias, all variables were still self-reported, and shared method variance cannot be completely ruled out. Future studies could incorporate multi-source data or objective behavioral indicators to more rigorously validate the proposed relationships. In addition, the sample size, while adequate for the basic requirements of a multi-wave longitudinal analysis, may still be constrained for detecting complex moderation effects. Future studies should employ larger samples to enhance statistical power.

Second, future research can further explore the heterogeneous effects of AI usage by functional categories. This study focuses on AI usage intensity due to the homogeneous technology context of China’s financial industry, but we acknowledge that AI tools with different functions may have divergent impacts on employees’ psychological processes. Subsequent studies can collect objective data on specific AI tool usage to classify AI functions more accurately, and test whether the dual-path mechanism varies across functional types.

Third, although this study examines dual mediating mechanisms and a key boundary condition, additional processes may also shape employees’ interpretations of AI. Future research could incorporate finer-grained psychological mechanisms or consider individual traits to further enrich the explanatory framework.

Finally, the sample was drawn from China’s financial industry, a context characterized by strong compliance demands, rapid technological change and specific demographic patterns. These features may limit the generalizability of the findings. Future work could examine whether the proposed model holds across different industries, technological environments and cultural contexts in order to better establish its external validity.

## Data Availability

The raw data supporting the conclusions of this article will be made available by the authors, without undue reservation.
